# Perinatal risk factors for congenital hypothyroidism: A retrospective cohort study performed at a tertiary hospital in China

**DOI:** 10.1097/MD.0000000000020838

**Published:** 2020-06-26

**Authors:** Jinfu Zhou, Jinying Luo, Junyu Lin, Yinglin Zeng, Xiaolong Qiu, Wenbin Zhu, Guanghua Liu

**Affiliations:** a Center of Neonatal Screening; b Department of Gynaecology and Obstetrics, Fujian Provincial Maternity and Child Hospital, Affiliated Hospital of Fujian Medical Universitya; c The First Affiliated Hospital of Fujian Medical University; d Department of Pediatrics, Fujian Provincial Maternity and Child Hospital, Affiliated Hospital of Fujian Medical University, Fuzhou, Fujian Province, China.

**Keywords:** congenital hypothyroidism, perinatal period, retrospective cohort study, risk factor

## Abstract

Congenital hypothyroidism (CH) is one of the most common neonatal endocrine diseases. This retrospective cohort study aimed to identify the potential perinatal risk factors for CH and to differentiate between transient and permanent CH (TCH and PCH, respectively) as well as determine their prevalence in a southeastern Chinese population.

This study was based on an 18-year surveillance of a neonatal CH screening program in a large tertiary hospital. A retrospective review of the maternal and neonatal perinatal exposures was conducted.

Of the 205,834 newborns screened between 2000 and 2018, 189 were diagnosed with CH (1/1089). Among the 131 CH patients who again underwent thyroid function testing (TFT) after discontinuation of levothyroxine at the age of 3 years, 61 (46.6%) were diagnosed with PCH and 70 (53.4%) were diagnosed with TCH. In the maternal characteristics model, women aged 35 years or older and those who had thyroid disease and/or diabetes mellitus during pregnancy had increased risk of having an offspring with CH (*P* = .001, .000, and .001, respectively). Significant associations were found with regard to parity and the risk of CH in the offspring (*P* = .000). In the neonatal characteristics model, infants with female sex, preterm birth, post-term birth, low birth weight, other birth defects, and those born as part of multiple births (*P* = .011, .034, .001, .000, .000, and .003, respectively) had increased risk of CH. The rate of newborns with other birth defects was higher in the PCH group than that in the TCH group (*P* = .008), whereas the rate of maternal thyroid disease, newborns with low birth weight, and newborns with preterm birth was higher in the TCH group than that in the PCH group (*P* = .041, .020, and .013, respectively). The levothyroxine dose (μg/kg/day) at 1 year, 2 years, and 3 years old was significantly lower in the TCH group than that in the PCH group (*P* = .000, .000, and .000, respectively).

Perinatal factors should be considered during the diagnosis and treatment of CH.

## 1 Introduction

Congenital hypothyroidism (CH) is one of the most common neonatal endocrine diseases, and with the increasing availability of neonatal screening programs, improved thyroid-stimulating hormone (TSH) assay sensitivity, and increased survival rate of preterm infants, the incidence of CH has increased worldwide.^[1,2]^ However, the etiology of CH has not been completely elucidated. Approximately 85% of CH cases are caused by thyroid dysgenesis, and the remaining 15% results from dyshormonogenesis. Most infants with CH at birth often have a normal appearance and nonspecific clinical symptoms and signs. If undiagnosed and untreated after birth, it can lead to growth failure and intellectual impairment, which are caused by the insufficient production of thyroid hormone. Screening programs for CH, which have been extensively carried out in many countries worldwide, provide the opportunity to investigate the epidemiology and etiology of CH. The incidence of CH in China is approximately 1 in 2000 live births.^[3]^

Although a variety of gene mutations have been involved in the mechanisms of CH,^[4,5]^ the occurrence of CH with genetic origin has been observed only in a relatively small proportion of patients. As CH mainly occurs in utero, maternal pregnancy complications, and abnormal fetal growth and development may be closely related to the condition. Some studies have recently shown that modifiable environmental risk factors contribute to the etiology of the disease,^[6,7]^ which hints at the possibility of preventing CH; hence, investigating those modifiable perinatal risk factors of CH is important.

Many CH cases are transient; they result from temporary abnormalities of thyroid hormone concentrations rather than permanent dysfunction. Current guidelines recommend that all infants with CH undergo levothyroxine treatment for up to 3 years old and have a repeat thyroid function test (TFT) to determine of the condition is permanent (PCH) or transient (TCH). A previous study has highlighted that overtreatment with thyroid hormone may have negative impact on developmental outcomes of infants.^[8]^ Thus, diagnosis and identification of and TCH are important to avoid unnecessary overtreatment as well as its possible side effects. However, the risk factors for TCH are not yet well established.

The current information about the relationship between perinatal factors and the incidence of CH is mainly based on case-control studies, but clinical information from large-sample-size studies has been rarely reported. To identify the potential perinatal risk factors for CH, we conducted a retrospective cohort study to search for maternal and neonatal exposure in Fujian Province, Southeast China. Furthermore, although previous Chinese studies have reported the proportion of TCH cases (from 19.9% to 55.5%),^[9–11]^ the rate of TCH in Fujian Province, Southeast China, is unknown; therefore, we also aimed to differentiate between TCH and PCH and determine their prevalence.

## 2 Materials and methods

### 2.1 Study design and data collection

This was a retrospective cohort study of maternal and neonatal perinatal exposure based on an 18-year surveillance of a neonatal CH screening program in a large tertiary hospital with 1500 beds in Fujian Province Maternity and Child Care Hospital, China. A total of 205,834 newborns under neonatal screening from January 1, 2000 to December 31, 2018, were included, and the subjects without neonatal screening were excluded. Neonatal screening tests (NSTs) were performed 3 to 7 days after birth. A dried heel prick blood sample was saved on filter paper, which was then processed at the laboratory of Fujian Province Maternity and Child Care Hospital. The level of TSH was determined using time-resolved immunofluorescence assay. Screen-positive cases of CH were divided into 2 thresholds based on TSH level: > 9 and > 18 μIU/ml. Patients with TSH above the thresholds were recalled, and the serum-free thyroxine (FT4) and TSH were measured from peripheral venous blood using electrochemiluninescence immunoassay (Roche Diagnostics Ltd, Switzerland). CH was diagnosed if the FT4 level was <0.9 ng/dl or if the TSH level was above the cutoff value (>20 μIU/ml at any time or > 10.0 μIU/ml after 4 weeks old).

Using a medical record system, we collected data on 11 types of information with the participants based on 2 categories: 1 was maternal reproductive and medical history, including demographic characteristics, gravidity, parity, gestational diabetes mellitus (GDM), thyroid disorder, and hypertensive disorder complicating pregnancy (HDCP); the other was neonatal characteristic, including sex, birth weight, gestational age, presence of other birth defects, multiple births, and fetal distress. If a newborn was diagnosed with CH, the TFT results and levothyroxine dosage information were also collected.

Levothyroxine treatment was performed after diagnosis of hypothyroidism. The initial levothyroxine dosage was 10 to 15 μg/kg/day, and it was adjusted according to follow-up TFT results. During the ages of 2.5 to 3 years, trial drug discontinuation was performed, and follow-up TFT were performed at 1, 6, and 12 months after levothyroxine discontinuation. Patients with normal TFT results for up to 12 months after discontinuation of levothyroxine were diagnosed with TCH. If the TFT showed FT4 level <0.9 ng/dl or TSH level >10.0 μU/ml, PCH was diagnosed, and levothyroxine was restarted.

### 2.2 Statistical analysis

Measurement data were described as mean ± standard deviation (SD) or median and percentiles for quantitative variables and as proportions for all categorical data. Differences in means or median were tested for statistical significance with independent-samples *t* test or Mann–Whitney test, and categorical data were compared with *χ*^2^ test or Fisher exact test. All statistical analyses were conducted with SPSS version 17.0 (IBM Corp., Armonk, NY, USA), and *P* < .05 was considered statistically significant.

## 3 Results

### 3.1 Clinical and laboratory characteristics of CH

From January 2000 to December 2018, 205,834 newborns were screened, and 2237 newborns had high TSH levels in the first dry sample. A total of 189 cases of CH were finally identified through confirmatory test, suggesting an overall incidence rate of 1 per 1089 screened newborns. Of the 189 subjects, 101 (53.4%) were female. The clinical and laboratory characteristics of CH are shown in Table 1.

**Table 1:**
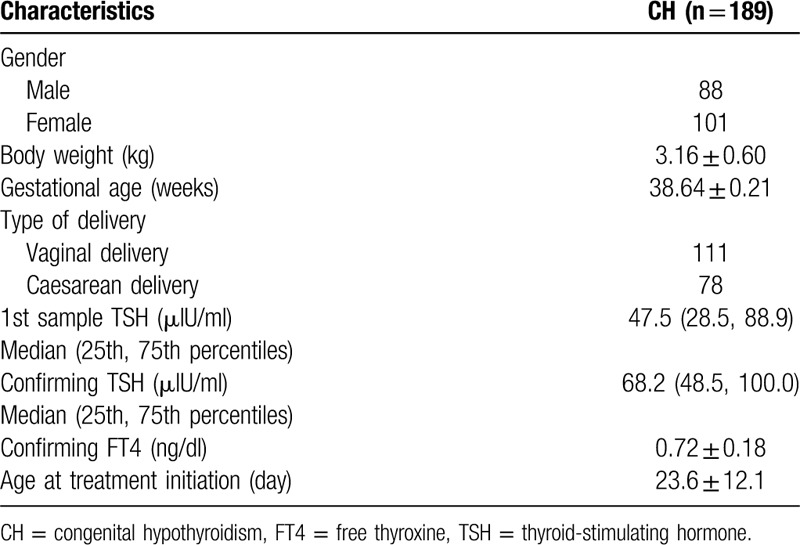
Clinical and laboratory characteristics of the congenital hypothyroidism.

### 3.2 Associations between maternal characteristics and the incidence of CH

In the maternal characteristics model, women aged 35 years or older and those who had thyroid disease and/or diabetes mellitus during pregnancy had increased risk of having an offspring with CH (OR = 2.007, 95% CI = 1.307–3.080, *P* = .001; OR = 3.558, 95% CI = 1.751–7.232, *P* = .000; OR = 2.167, 95% CI = 1.348–3.483, *P* = .001, respectively. Significant associations were observed between parity and the risk of CH in the offspring (*χ*^*2*^_trend_ = 52.78, *P* = .000). However, no significant associations were observed between gravidity or HDCP and the risk of CH in the offspring (Table 2).

**Table 2:**
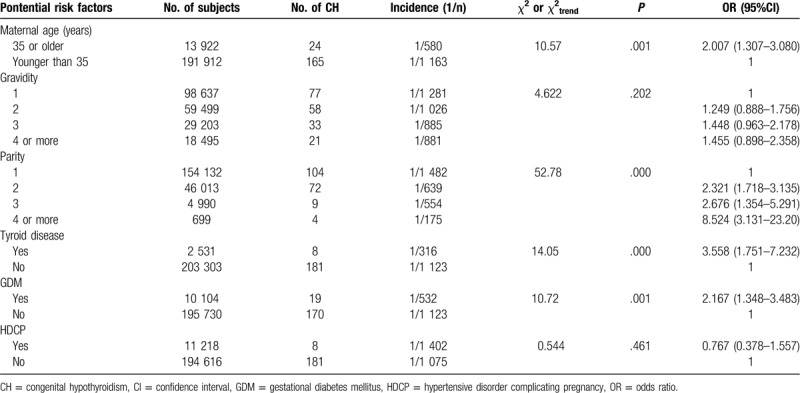
Associations between maternal characteristics and the incidence of CH.

### 3.3 Associations between neonatal characteristics and the incidence of CH

In the neonatal characteristics model, infants with female sex (OR = 1.446, 95% CI = 1.087–1.925, *P* = .011), preterm birth (OR = 1.506, 95% CI = 1.029–2.204, *P* = .034), post-term birth (OR = 3.824, 95% CI = 1.571–9.305, *P* = .001), low birth weight (OR = 2.628, 95% CI = 1.815–3.806, *P* = .000), other birth defects (OR = 4.697, 95% CI = 3.127–7.054, *P* = .000), and those born as part of multiple births (OR = 2.102, 95% CI = 1.278–3.459, *P* = .003) had increased risk of CH. However, no significant association was observed between fetal distress and the risk of CH (Table 3).

**Table 3:**
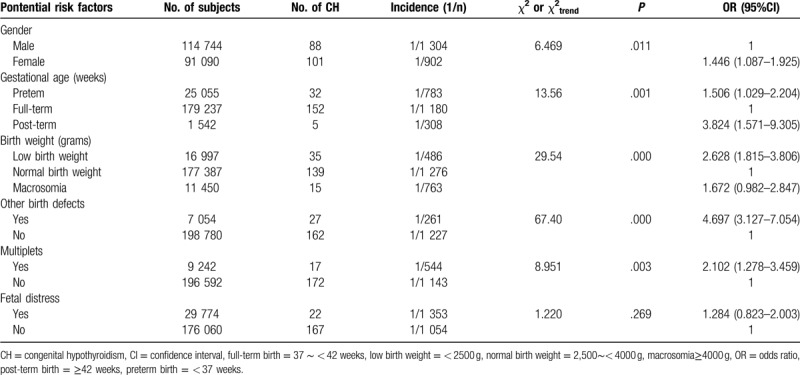
Associations between neonatal characteristics and the incidence of CH.

### 3.4 Clinical and laboratory characteristics of PCH and TCH

A total of the 131 subjects (69 female and 62 male) underwent trial discontinuation. Discontinuation of replacement treatment failed in 61 infants (46.6%), and they were diagnosed with PCH (PCH group), whereas the rest (70, 53.4%) who successfully discontinued replacement treatment were diagnosed with TCH (TCH group). The rate of maternal thyroid disease and newborns with other birth defects showed significant differences between the TCH and PCH groups (*P* = .041 and .008, respectively) (Table 4). Although birth weight and gestational age showed no differences between the TCH and PCH groups, the rates of low birth weight and preterm birth were higher in the TCH group than in the PCH group (*P* = .020 and .013) (Table 4). The initial NST and confirmatory TSH levels were significantly higher in the PCH group than those in the TCH group (*P* = .041 and .008), but the confirmatory FT4 level exhibited no differences between the 2 groups (*P* = .196) (Table 4). The levothyroxine doses (μg/kg/day) at 1 year, 2 years, and 3 years old were significantly lower in the TCH group than those in the PCH group (*P* = .000,.000, and .000, respectively). However, the subjects age at the time of treatment initiation and the initial levothyroxine dose showed no significant differences between the TCH and PCH groups (Table 4).

**Table 4:**
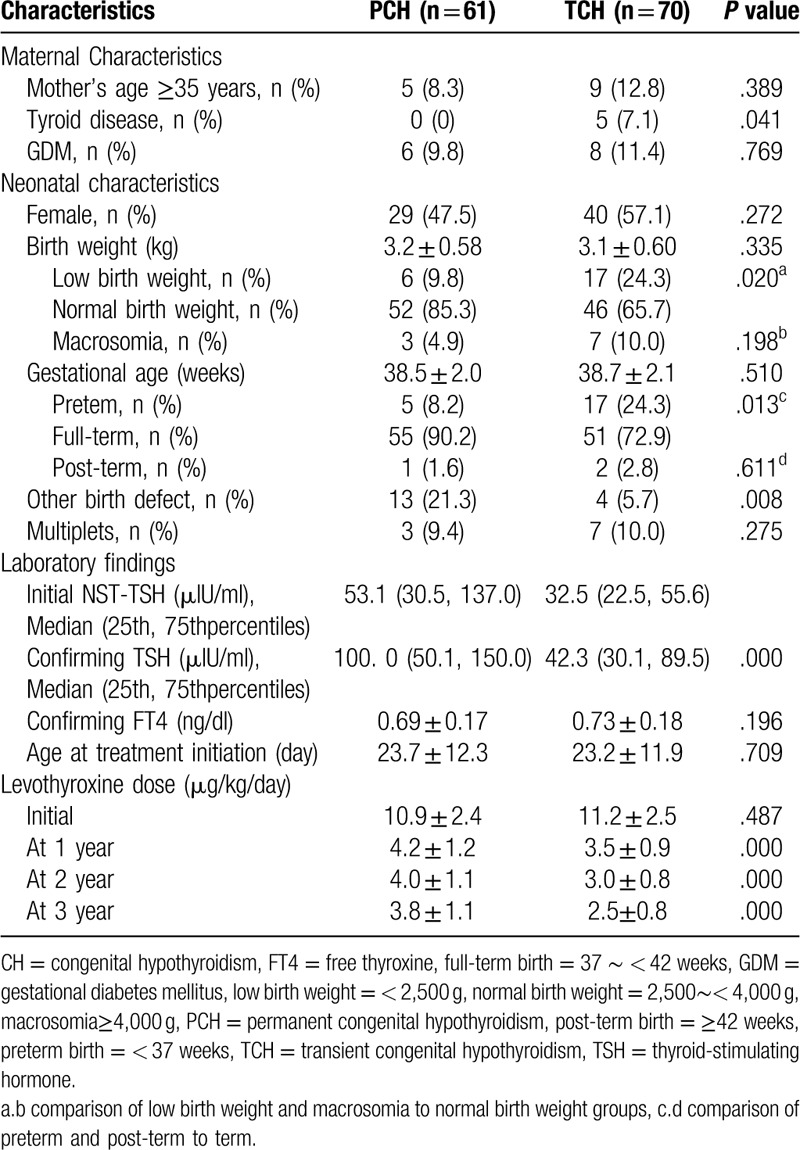
Clinical and laboratory characteristics of permanent & transient congenital hypothyroidism.

## 4 Discussion

In this study, we identified the potential perinatal risk factors for CH and differentiated between TCH and PCH as well as determined their prevalence in Fujian Province, Southeast China. In this study, the overall incidence of CH was 1/1089, and 53.4% of the total 131 patients with CH were diagnosed with TCH. Maternal perinatal factors such as advanced maternal age and gestational complications and neonatal perinatal factors such as female sex, preterm birth, postnatal birth, low birth weight, presence of other birth defects, and being born as part of multiple births were closely related to the occurrence of CH.

Complications of pregnancy, such as GDM, hyperthyroidism, and HDCP, can increase the risk of short- and long-term adverse effects in both the mother and the offspring.^[12,13]^ The available antithyroid drugs given to treat maternal hyperthyroidism during pregnancy can cross the placenta, which may cause TCH.^[14]^ Aside from antithyroid drugs, additional factors can potentially disrupt fetal thyroid function. In infants of mother with autoimmune thyroid disease, maternal TSH receptor antibodies that block the activation of the TSH receptor can be transported across the placenta and cause fetal hypothyroidism.^[15]^ In this study, we found that women who had thyroid disease during pregnancy had a higher risk of having an offspring with CH, and the rate of maternal thyroid disease was higher in the TCH group than that in the PCH group, which was similar to the findings of a previous study.^[16]^ Our results also showed that GDM increases the risk of CH in the offspring, which was consistent with previous report.^[16]^ In an animal model of GDM, the degree of diabetes mellitus was negatively correlated with the fetal thyroid hormone status.^[17]^ In addition, there was an increased risk of subclinical hypothyroidism during GDM.^[18]^ However, the causal relationship between GDM and CH in the offspring is unclear. Our results showed that the rate of GDM was not different between the TCH and PCH groups, which was not similar to the results of a previous study.^[16]^

Advanced maternal age and multiparity are high-risk factors for birth defects.^[19,20]^ In this study, advanced maternal age and multiparity increased the risk of CH in the offspring. In women with advanced maternal age, the decline in physiological and reproductive functions can lead to adverse pregnancy outcomes. Multiparous mothers have increased risk of genital tract infection, which may affect the environment and nutrition of the embryo.

Our study confirmed a significantly higher prevalence of CH in female than in male offspring. However, underlying causes of the difference are unclear. Thyroid dysgenesis is the leading cause of PCH, and the high female-to-male ratio among cases of CH is mostly associated with dysgenesis of the thyroid gland.^[21]^ However, a recent study showed that female preponderance is not seen in patients with dysgenesis of the thyroid gland.^[22]^ Our study also confirmed that the female-to-male ratio was not difference between the TCH and PCH groups. Further study should be carried out to demonstrate whether a female preponderance is associated with PCH. Our study found that the occurrence of CH was closely related to gestational age and birth weight, which was consistent with previous studies.^[23,24]^ Preterm birth is a significant risk factor for CH with eutopic thyroid. Infants with low birth weight and preterm birth are at an increased risk for intrauterine dysplasia, resulting in hypothalamic-pituitary-thyroid dysfunction.^[24]^ In addition, infants with preterm birth and low birth weight are at elevated risk of iodine deficiency, in part because the preterm infant formulas and parenteral nutrition commonly used in intensive care settings may not provide adequate iodine,^[25]^ and Iodine deficiency can lead to CH. It is still unclear why infants with advanced gestational age (≥42 weeks) are more susceptible to developing CH. In the current study, we found that gestational age and birth weight were not significantly different between the TCH and PCH groups. However, the rates of low birth weight and preterm birth were higher in the TCH group, and further research is needed to confirm the relationships. Twins had increased risk for either PCH or TCH.^[16]^ Twins are frequently born preterm and/or have low birth weight, which can impair the function of the hypothalamic-pituitary-thyroid axis. A recent study on twins with CH showed that 64% common environmental (shared during the fetal life) and genetic factors and 34% unshared environmental factors (36%) are responsible in PCH, whereas 95% genetic factors and 5% unshared environmental factors are responsible for TCH.^[26]^ As expected,^[27,28]^ in our study, infants with other birth defects had increased risk of CH, and the PCH group had a higher frequency of malformations than the TCH group. It was reported that a group of cases with CH associated with other birth defects had higher prevalence of thyroid agenesis than the group with no other birth defects.^[29]^ Hence, it is important to investigate the molecular mechanisms underlying the events of the abnormal formation of the thyroid and other organs. The paired box gene 8 (*PAX8*), which encodes 1 family of 9 transcription factors in humans, plays a crucial role in embryogenesis, especially in the thyroid gland.^[30]^ Interestingly, a previous study showed heterozygous *PAX8* mutation was associated with urogenital malformations in CH patients.^[31]^ Homozygous mutations in thyroid transcription factor 2 were confirmed in patients with a syndromic form of CH.^[32]^*NKX2–5* gene plays an important role in heart development,^[33]^ and mutations in the *NKX2–5* gene have been found in CH cases with ectopy or athyreosis.^[34]^ Despite recent progress, the relationship between malformations and CH remains to be elucidated.

In our study, 53.4% of the cases with CH were diagnosed with TCH, which was similar to the results of previous Chinese studies.^[9,10]^ To maintain normal thyroid function, the dose of levothyroxine required in the PCH group was higher than that in the TCH group.^[35,36]^ Several studies suggested that different doses of levothyroxine required during treatment or at discontinuation might be used to predict PCH.^[35,37,38]^ In the current study, the levothyroxine doses at 1 year, 2 years, and 3 years old were significantly higher in the PCH group than those in the TCH group, which was similar to the findings of previous studies.^[35,38]^ Whether laboratory findings can predict TCH is controversial. A significant reduction in confirmatory TSH levels in infants with TCH compared to infants with PCH was reported.^[38]^ However, other studies have shown no difference in the confirmatory FT4 and TSH levels between cases with TCH and PCH.^[35,37]^ In the current study, the initial NST and confirmatory TSH levels in the TCH group were significantly lower than those in the PCH group, but there was no difference in the FT4 between the 2 groups.

The limitations of our study were its retrospective design and the lack of analysis of other potential risk factors such as genetic susceptibility and environmental exposure. It is also important to investigate the cognitive function and growth outcomes of patients with CH. Nevertheless, our study had some advantages. It has a relatively large number of infants. Furthermore, this was a single-center study, which is conducive to relatively consistent quality control of disease diagnosis and treatment.

In conclusion, the results of the current study have shown that maternal and neonatal perinatal factors contribute to the etiology of CH. Low birth weight and preterm birth are risk factors for TCH, whereas the presence of other birth defects is closely related to PCH. These results provide insights into the role of perinatal factors in the pathogenesis of CH and in the treatment of CH. Notably, maternal and neonatal perinatal factors should be considered during the diagnosis and treatment of CH. In future studies, we will explore and investigate other potential causes of CH, such as susceptible genes, environmental factors, and epigenetic characteristics.

## Author contributions

JZ, JL, WZ and GL conceived and designed the study; JZ, JuL, YZ and XQ carried out this study; JZ, JL, JuL, YZ and XQ analyzed the data of this study; JZ and JL wrote the paper; JZ, WZ and GL reviewed and edited the manuscript. All authors read and approved the manuscript.

## References

[1] Barry Y Bonaldi C Goulet V Increased incidence of congenital hypothyroidism in France from 1982 to 2012: a nationwide multicenter analysis. Ann Epidemiol 2016;26:100–5.e4. 26775052 10.1016/j.annepidem.2015.11.005

[2] Olivieri A Fazzini C Medda E Multiple factors influencing the incidence of congenital hypothyroidism detected by neonatal screening. Hormone Res Paediatr 2015;83:86–93. 10.1159/000369394 25572470

[3] Deng K He C Zhu J Incidence of congenital hypothyroidism in China: data from the national newborn screening program 2013-2015. J Pediatr Endocrinol Metab 2018;31:601–8. 29715190 10.1515/jpem-2017-0361

[4] Löf C Patyra K Kuulasmaa T Detection of novel gene variants associated with congenital hypothyroidism in a finnish patient cohort. Thyroid 2016;26:1215–24. 27373559 10.1089/thy.2016.0016 PMC5036323

[5] Rurale G Persani L Marelli F GLIS3 and thyroid: a pleiotropic candidate gene for congenital hypothyroidism. Front Endocrinol 2018;9 (November):1–7. 10.3389/fendo.2018.00730 PMC6281699 30555422

[6] Shang L Huang L Yang W Maternal exposure to PM2.5 may increase the risk of congenital hypothyroidism in the offspring: a national database based study in China. BMC Public Health 2019;19:1412. 31739791 10.1186/s12889-019-7790-1 PMC6862828

[7] Kim DH Kim UJ Kim HY Perfluoroalkyl substances in serum from South Korean infants with congenital hypothyroidism and healthy infants - Its relationship with thyroid hormones. Environ Res Elsevier 2016;147:399–404. 10.1016/j.envres.2016.02.037 26950028

[8] Jones JH Gellén B Paterson WF Effect of high versus low initial doses of L-thyroxine for congenital hypothyroidism on thyroid function and somatic growth. Arch Dis Childhood 2008;93:940–4. 18456702 10.1136/adc.2007.120618

[9] Yang HH Qiu L Zhao JQ Epidemiologic characteristics and risk factors for congenital hypothyroidism from 1989 to 2014 in Beijing. Chin J Prv Med 2016;50:728–32. 10.3760/cma.j.issn.0253-9624.2016.08.011 27539527

[10] Fu C Luo S Li Y The incidence of congenital hypothyroidism (CH) in Guangxi, China and the predictors of permanent and transient CH. Endocr Connect 2017;6:926–34. 29074613 10.1530/EC-17-0289 PMC5704446

[11] Chen J Lin S Zeng G Epidemiologic characteristics and risk factors for congenital hypothyroidism from 2009 to 2018 in Xiamen, China. Endocr Pract 2020;doi: 10. 4158/EP-2019-0491. 10.4158/EP-2019-0491 31968198

[12] McIntyre HD Catalano P Zhang C Gestational diabetes mellitus. Nat Rev Dis Primers, Springer US 2019;5.:10.1038/s41572-019-0098-8 31296866

[13] Moleti M Di Mauro M Sturniolo G Hyperthyroidism in the pregnant woman: maternal and fetal aspects. J Clin Transl Endocrinol Elsevier 2019;16 (March):100190. 10.1016/j.jcte.2019.100190 PMC6484219 31049292

[14] Bliddal S Rasmussen ÅK Sundberg K Antithyroid drug-induced fetal goitrous hypothyroidism. Nat Rev Endocrinol Nature Publishing Group 2011;7:396–406. 10.1038/nrendo.2011.34 21403664

[15] Wassner AJ Congenital hypothyroidism. Clin Perinatol Elsevier Inc 2018;45:1–8. 10.1016/j.clp.2017.10.004 29405999

[16] Medda E Olivieri A Stazi MA Risk factors for congenital hypothyroidism: results of a population case-control study (1997-2003). Euro J Endocrinol 2005;153:765–73. 10.1530/eje.1.02048 16322381

[17] Calvo R Morreale De Escobar G Escobar Del Rey F Maternal nonthyroidal illness and fetal thyroid hormone status, as studied in the streptozotocin-induced diabetes mellitus rat model. Endocrinology 1997;138:1159–69. 9048623 10.1210/endo.138.3.4997

[18] Olivieri A Valensise H Magnani F High frequency of antithyroid autoantibodies in pregnant women at increased risk of gestational diabetes mellitus. Euro J Endocrinol 2000;143:741–7. 10.1530/eje.0.1430741 11124856

[19] Jaruratanasirikul S Chicharoen V Chakranon M Population-based study of prevalence of cleft lip/palate in southern Thailand. Cleft Palate-Craniofacial J 2016;53:351–6. 10.1597/14-259 26406558

[20] Mcneese ML Selwyn BJ Duong H The association between maternal parity and birth defects. Birth Defects Res Part A 2015;103:144–56. 10.1002/bdra.23360 25721953

[21] Castanet M Polak M Léger J Familial forms of thyroid dysgenesis. Endocr Develop 2007;10:15–28. 10.1159/000106817 17684387

[22] Dayal D Sindhuja L Bhattacharya A Agenesis and not ectopia is common in North Indian children with thyroid dysgenesis. Indian J Endocrinol Metab 2014;18:S97–9. 25538886 10.4103/2230-8210.145080 PMC4266877

[23] Hashemipour M Hovsepian S Ansari A Screening of congenital hypothyroidism in preterm, low birth weight and very low birth weight neonates: a systematic review. Pediatr Neonatol, Taiwan Pediatr Assoc Elsevier Taiwan LLC 2018;59:3–14. 10.1016/j.pedneo.2017.04.006 28811156

[24] Kaluarachchi DC Allen DB Eickhoff JC Increased congenital hypothyroidism detection in preterm infants with serial newborn screening. J Pediatr Elsevier Inc 2019;207:220–5. 10.1016/j.jpeds.2018.11.044 30579585

[25] Belfort MB Pearce EN Braverman LE Low iodine content in the diets of hospitalized preterm infants. J Clin Endocrinol Metab 2012;97:632–6. 22337912 10.1210/jc.2011-3369 PMC3319182

[26] Medda E Vigone MC Cassio A Neonatal screening for congenital hypothyroidism: what can we learn from discordant twins. J Clin Endocrinol Metab 2019;104:5765–79. 31287502 10.1210/jc.2019-00900

[27] Örnek N O⊠urel R Örnek K Congenital hypothyroidism in Rieger syndrome. Ophthal Genet 2016;37:86–8. 10.3109/13816810.2014.902079 24666291

[28] Kumar J Gordillo R Kaskel FJ Increased prevalence of renal and urinary tract anomalies in children with congenital hypothyroidism. J Pediatr 2009;154:263–6. 18823909 10.1016/j.jpeds.2008.08.023 PMC3749842

[29] Monroy-Santoyo S Ibarra-González I Fernández-Lainez C Higher incidence of thyroid agenesis in Mexican newborns with congenital hypothyroidism associated with birth defects. Early Hum Develop 2012;88:61–4. 10.1016/j.earlhumdev.2011.07.009 21816548

[30] De Felice M Di Lauro R Minireview: intrinsic and extrinsic factors in thyroid gland development: an update. Endocrinology 2011;152:2948–56. 21693675 10.1210/en.2011-0204

[31] Carvalho A Hermanns P Rodrigues AL A new PAX8 mutation causing congenital hypothyroidism in three generations of a family is associated with abnormalities in the urogenital tract. Thyroid 2013;23:1074–8. 23647375 10.1089/thy.2012.0649

[32] Abu-Khudir R Larrivée-Vanier S Wasserman JD Disorders of thyroid morphogenesis. Best Pract Res 2017;31:143–59. 10.1016/j.beem.2017.04.008 28648504

[33] Benaglio P D’Antonio-Chronowska A Ma W Allele-specific NKX2-5 binding underlies multiple genetic associations with human electrocardiographic traits. Nat Genet 2019;51:1506–17. 31570892 10.1038/s41588-019-0499-3 PMC6858543

[34] Targovnik HM Scheps KG Rivolta CM Defects in protein folding in congenital hypothyroidism: protein folding in congenital hypothyroidism. Mol Cell Endocrinol 2020;501. 10.1016/j.mce.2019.110638 31751626

[35] Park ES Yoon JY Factors associated with permanent hypothyroidism in infants with congenital hypothyroidism. BMC Pediatr 2019;19:453. 31752783 10.1186/s12887-019-1833-8 PMC6873549

[36] Oron T Lazar L Ben-Yishai S Permanent vs transient congenital hypothyroidism: assessment of predictive variables. J Clin Endocrinol Metab 2018;103:4428–36. 30272179 10.1210/jc.2018-00362

[37] Rabbiosi S Vigone MC Cortinovis F Congenital hypothyroidism with eutopic thyroid gland: analysis of clinical and biochemical features at diagnosis and after re-evaluation. J Clin Endocrinol Metab 2013;98:1395–402. 23426615 10.1210/jc.2012-3174

[38] Park IS Yoon JS So CH Predictors of transient congenital hypothyroidism in children with eutopic thyroid gland. Ann Pediatr Endocrinol Metab 2017;22:115. 28690990 10.6065/apem.2017.22.2.115 PMC5495977

